# The Effect of Exposed Glass Fibers and Particles of Bioactive Glass on the Surface Wettability of Composite Implants

**DOI:** 10.1155/2011/607971

**Published:** 2011-12-27

**Authors:** Aous A. Abdulmajeed, Lippo V. Lassila, Pekka K. Vallittu, Timo O. Närhi

**Affiliations:** ^1^Department of Prosthetic Dentistry, Institute of Dentistry, University of Turku, Lemminkäisenkatu 2, 20520 Turku, Finland; ^2^Turku Clinical Biomaterials Centre (TCBC), University of Turku, 20520 Turku, Finland; ^3^Finnish Doctoral Program in Oral Sciences (FINDOS), Institute of Dentistry, University of Turku, 20520 Turku, Finland; ^4^Department of Biomaterials Science, Institute of Dentistry and BioCity Turku Biomaterial Research Program, University of Turku, 20520 Turku, Finland; ^5^Turku University Centre for Materials and Surfaces (MatSurf), University of Turku, 20520 Turku, Finland; ^6^Clinic of Oral Diseases, Turku University Central Hospital, 20520 Turku, Finland

## Abstract

Measurement of the wettability of a material is a predictive index of cytocompatibility. This study was designed to evaluate the effect of exposed E-glass fibers and bioactive glass (BAG) particles on the surface wettability behavior of composite implants. Two different groups were investigated: (a) fiber reinforced composites (FRCs) with different fiber orientations and (b) polymer composites with different wt. % of BAG particles. Photopolymerized and heat postpolymerized composite substrates were made for both groups. The surface wettability, topography, and roughness were analyzed. Equilibrium contact angles were measured using the sessile drop method. Three liquids were used as a probe for surface free energy (SFE) calculations. SFE values were calculated from contact angles obtained on smooth surfaces. The surface with transverse distribution of fibers showed higher (*P* < 0.001) polar (*γ* ^P^) and total SFE (*γ* ^TOT^) components (16.9 and 51.04 mJ/m^2^, resp.) than the surface with in-plane distribution of fibers (13.77 and 48.27 mJ/m^2^, resp.). The increase in BAG particle wt. % increased the polar (*γ* ^P^) value, while the dispersive (*γ* ^D^) value decreased. Postpolymerization by heat treatment improved the SFE components on all the surfaces investigated (*P* < 0.001). Composites containing E-glass fibers and BAG particles are hydrophilic materials that show good wettability characteristics.

## 1. Introduction

The surface properties of an implant and its prosthetic abutments provide one of the most important conditions relating to the future success of oral implant procedures [1]. Since these components penetrate through the gingival tissues and are exposed to the oral cavity, they play an important role not only in biocompatibility but also in bacterial adhesion and stagnation [2].

Biomaterial surface quality can be measured by a combination of physical, chemical, and mechanical properties and its surface structure [3]. It is generally accepted that early surface events that occur rapidly upon implantation of a biomaterial into biological fluids determine the subsequent responses. These involve wetting by physiological liquids, followed by adsorption of proteins and cells to the biomaterial surface [4].

Cellular behaviors such as adhesion, morphological change, functional alteration, and proliferation are greatly affected by surface properties such as hydrophilicity, roughness, charge, free energy, and morphology [5–7]. It is well known that surface chemistry, surface energy, and surface topography govern the biological response to an implant material [8]. Hallab et al. [9] demonstrated that surface free energy (SFE) was a more important surface characteristic than surface roughness for cellular adhesion strength and proliferation and that the SFE components of the various materials tested were shown to be related to cellular adhesion strength. Schakenraad et al. [10] found that despite the great number of parameters interfering with cellular adhesion and spreading, solid SFE is apparently a dominant factor in cellular attachment to a polymer surface and remains so, even if the solid surface has been covered by a protein layer. It has been reported that roughness could disturb the relationships between SFE and cell proliferation [11]. However, it is postulated that the influences of surface roughening on contact angles disappear if the average surface roughness (*R*_*a*_) is <0.1 *μ*m [12].

**Figure 1:**
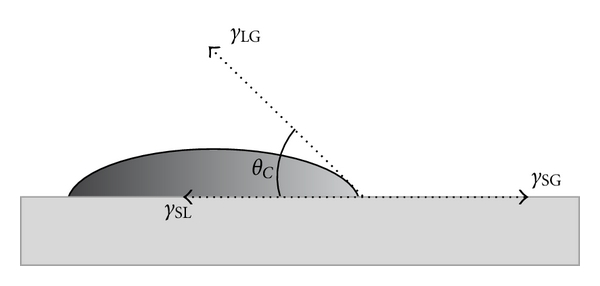
An illustration of the sessile drop technique with a liquid droplet partially wetting a solid substrate: *θ*_*C*_: equilibrium contact angle; *γ*_SG_: interfacial tension between the solid and gas; *γ*_SL_: interfacial tension between the solid and liquid; *γ*_LG_: interfacial tension between the liquid and gas.

Measurement of the wettability of a material, expressed by the contact angle (*θ*_*C*_) in the presence of the different liquids, is a predictive index of cytocompatibility [1, 13]. Contact angle techniques evolved from the method first described by Young in 1805, as cited by Baier et al. [14]. If a small amount of liquid is deposited on a solid surface and it does not spread, a drop is formed. The angle of intersection of a line tangent to the liquid and the surface of the solid that it contacts is the contact angle (Figure 1). This angle is characteristic of the substances in the system because of the surface tension of the liquid and the surface energy of the solid, modified by certain properties such as roughness. Stated another way, a low contact angle indicates good wettability, whereas a high contact angle results in poor wettability [15]. Thus, the relative wetting characteristics of a liquid-solid interface can be inferred by contact angle measurements.

SFE is important for wettability [14]. Surface energy is the energy at the surface of a solid, which is greater than its interior energy. The outer atoms are not equally attracted to each other as in the inner layer of atoms. The energy is greatest on the outermost atomic layer because the unsaturated bonds generate surface energies [17]. SFE can be determined by measuring the contact angles formed by a range of liquids on a given surface, using several diverse approaches [18]. When a liquid is placed on a lower-energy surface, the contact angle will be higher as compared with a higher-energy surface [14].

The use of fiber reinforced composite (FRC) has increased in many dental applications [19–22]. Recently, attempts have been made to use FRC as implant material in dental, orthopedic, and craniofacial surgeries [23–25]. Although there is a limited amount of scientific literature on using FRC material as surgical devices, they show potential for their use in surgery, especially when combined with bioactive modifiers, such as bioactive glass (BAG).

Bioactive materials such as BAGs interact with body fluids, and the subsequent formation of a calcium-phosphate rich (Ca-P) layer on their surface allows them to bond tightly to hard and soft connective tissues [26–28]. The attachment mechanism is associated with the development of a carbonate-containing hydroxyapatite layer on the surface of the materials, which provides a strong and stable connection with living tissues [26–29]. BAG was selected as it promotes cell attachment by soft tissues on the surface of implants [29] and induces periodontal tissue attachment [28].

The release of residual monomers from BisGMA-TEGDMA polymer may influence the biocompatibility of polymer implants [30]. Because of this, the composite implants should have an optimum degree of monomer conversion (DC %). This can be obtained by lengthening the photopolymerization time in combination with postpolymerization at increased temperature [31].

The aim of this study was to evaluate the surface average roughness (*R*_*a*_), topography, and wettability provided by different E-glass fiber orientations and BAG particles percentages through the combined use of contact angle meter and confocal microscopy. This study is based on the working hypothesis that addition of E-glass fibers and BAG particles to the composite matrix influences the wettability behavior of the surfaces investigated.

## 2. Materials and Methods

### 2.1. Study Design

**Figure 2:**
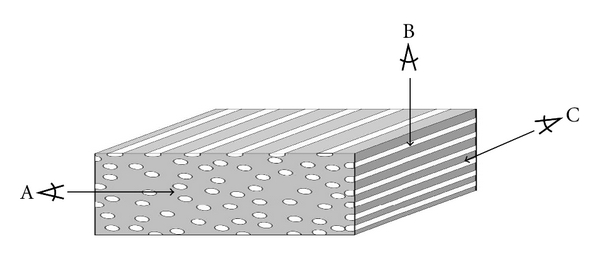
Schematic and simplified picture of FRC specimen showing fibers in different orientation planes: (A) fibers running transversely; (B) fibers running in-plane (perpendicular); (C) Fibers running in-plane (parallel).

Two different groups of substrates were investigated in the present study (*n* = 10 per group). The first group was composed of unidirectional FRCs of different fiber orientations (transverse and in-plane directions) (Figure 2). The second group was composed of polymer composites with increasing BAG particle percentages (25%, 50%, 75%, and 100% by weight, resp.). Plain polymer and plain BAG substrates were used as negative and positive controls. For both groups, two different sets were prepared with different curing methods (photopolymerized and postpolymerized of increased temperature).

The final substrate cutting procedure exposed the glass-fibers and BAG particles on the substrate surfaces. The different surfaces were characterized by means of surface roughness (*R*_*a*_) determinations, contact angle measurements, and SFE calculations.

### 2.2. Specimen Preparation

The materials used for the fabrication of the specimens for this study are listed in Table 1. E-glass fibers and BAG particles were mixed with acrylates, cast into molds (10 × 10 × 2 mm), and photopolymerized for 60 seconds (Elipar S10, 3M Espe, Germany). The irradiance was 950 mW/cm^2^, as measured with a curing radiometer. Subsequently, to optimize the DC %, one set of specimens was postpolymerized in an oven for 24 hours at 120°C. The unidirectional silane-sized E-glass fiber rovings were resin impregnated with light-polymerizable BisGMA-TEGDMA (50–50%) resin and incubated in an incubator at 37°C for 48 hours (D 06062, Modell 600, Memmert GmbH + Co. KG, Deutschland). The composition of E-glass fibers by weight is 55% SiO_2_, 15% Al_2_O_3_, 22% CaO, 6% B_2_O_3_, 0.5% MgO, and >1.0% Fe + Na + K. The composition of BAG particles by weight is SiO_2_ 53%, Na_2_O 23%, CaO 20%, and P_2_O_5_ 4%.

The surfaces of the specimens were ground using wet silicon carbide grinding paper (FEPA #4000) and polishing cloths with a 0.1 *μ*m AP-D suspension using a grinding machine (LaboPol-21, Struers A/S, Denmark). This polishing procedure ensures that average surface roughness (*R*_*a*_) is lower than 0.1 *μ*m, to eliminate the effect of surface roughness on contact angle measurements. After each polishing step, the specimens were ultrasonically cleaned in distilled water for 10 minutes to remove possibly embedded grinding material. Specimens were retained after each polishing step for contact angle measurements and *R*_*a*_ determination. The specimens were conditioned at room temperature for 2 days before testing.

### 2.3. Roughness and Imaging Characterization

A spinning disk confocal microscope with a white light source (COM, NanoFocus *μ*Surf, Germany) was used to determine 3D surface parameters. The average 3D surface roughness value (*R*_*a*_) according to DIN EN ISO 4287 was measured at 100x magnification. At 100x magnification, the vertical resolution of the lens is 2 nm and the numerical aperture 0.8–0.95 for a measurement area of 160–158 *μ*m. For the measurements done with the 100x lens, the cutoff wavelength of 80 *μ*m was used. Six readings per specimen were made at different randomly chosen locations. The mean value was calculated and quantitatively expressed as a numerical value (in microns) and a graph of the profile.

### 2.4. Contact Angle Measurements

Equilibrium contact angles (*θ*_*C*_) were measured using the sessile drop method (described in detail elsewhere [32]) with a contact angle meter (KSVCAM100 KSV, Instruments LTD, Finland). A drop was deposited on the surface and imaged for 20 seconds by collecting one image per 2 seconds. Determination of contact angle was based on the Young-Laplace equation, yielding the contact angles on both sides of the droplet and their mean values. Three liquids were used as a probe for SFE calculations (Table 2). The result was the mean of at least 10 drops on each specimen. Regarding the specimens that contain E-glass fibers, the contact angles were measured in two directions with respect to fiber orientation (parallel and perpendicular to the camera's axis). A total of 5,700 measurements were taken.

### 2.5. Surface Free Energy Calculations

The SFE was calculated using two theoretical models, the Owens-Wendt (OW) and Van-Oss (VO) approaches. The OW model approach gives the long-range dispersion (Lifshitz-van der Waals) (*γ* ^D^) and the short-range polar (hydrogen bonding) (*γ* ^P^) components of SFE [33], and the VO approach brings the dispersive (*γ* ^LW^) and the polar acid-base (*γ* ^AB^) components, the latter divided into two parts, acidic (*γ* ^+^) and basic (*γ* ^−^) [34] according to the following equations:

(1) $$\begin{array}{cc} 1+\cos\theta & =2{\left(\gamma_{\text{s}}^{\text{d}}\right)}^{1/2}\left(\frac{{\left(\gamma_{\text{L}}^{\text{d}}\right)}^{1/2}}{\gamma_{\text{L}}}\right)+2{\left(\gamma_{\text{s}}^{\text{p}}\right)}^{1/2}\left(\frac{{\left(\gamma_{\text{L}}^{\text{P}}\right)}^{1/2}}{\gamma_{\text{L}}}\right),\\ {\left(1+\cos\theta \right)}_{\gamma_{\text{L}}} & =2\left[{\left(\gamma_{\text{s}}^{\text{LW}}\gamma_{\text{L}}^{\text{LW}}\right)}^{1/2}+{\left(\gamma_{\text{L}}^{-}\gamma_{\text{s}}^{+}\right)}^{1/2}+{\left(\gamma_{\text{s}}^{-}\gamma_{\text{L}}^{+}\right)}^{1/2}\right], \end{array}$$

where *γ*_s_ is the SFE of the surface, *γ*_L_ the SFE of the liquid, and *γ*_s_^ab^ = (*γ*_s_^−^ *γ*_L_^+^)^1/2^.

For both methods, the spreading pressure was not taken into account. This pressure contributes to SFE and has to be considered if the SFE is higher than 60 mJ/m^2^ [35]. In the present work, SFE values were lower than this limit and the spreading pressure can be disregarded.

### 2.6. Statistical Analysis

Statistical analyses were performed using the SPSS v.14.0 software package (SPSS Inc.). Analysis of variance (ANOVA) followed by Tukey's post-hoc test was used to analyze the differences among several means. Differences were considered significant at a 95% confidence level. The independent variable was the contact angle, and dependant variables were fiber orientation and quantity of BAG in the composite, and the type of polymerization.

## 3. Results

### 3.1. Roughness and Imaging Characterization

**Figure 3:**
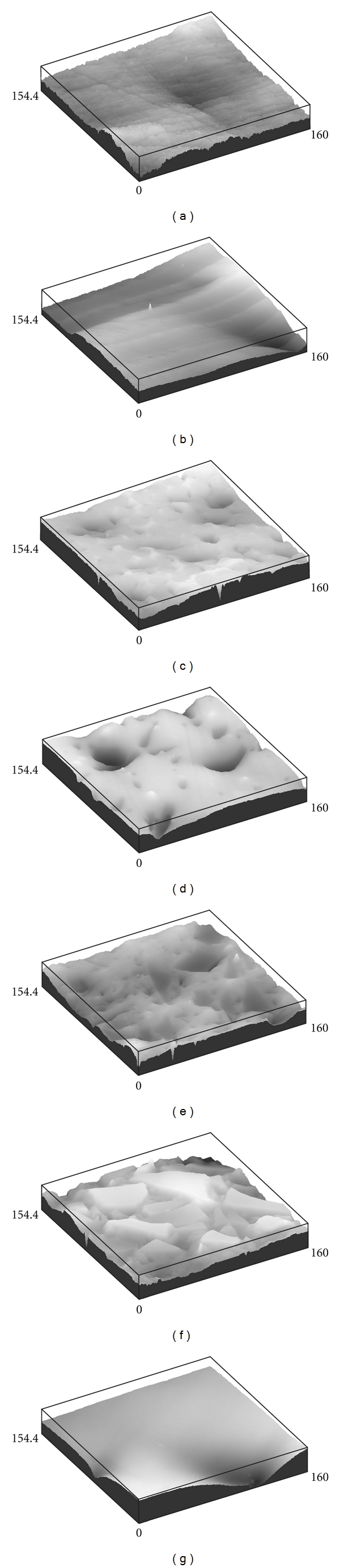
Confocal profiler 3D images of specimens investigated: (a) 100% polymer; (b) polymer with in-plane oriented fibers; (c) polymer with transversely oriented fibers; (d) 75% polymer + 25 wt. % fraction of BAG-particles; (e) 50% polymer + 50 wt. % fraction of BAG-particles; (f) 25% polymer + 75 wt. % fraction of BAG-particles; (g) 100% BAG.

The average surface roughness (*R*_*a*_) obtained through confocal microscopy measurements is presented in Table 3. All surfaces investigated showed *R*_*a*_ values of less than 0.1 *μ*m. The confocal profiler 3D images of the investigated specimens after smooth polishing are shown in Figure 3.

### 3.2. Contact Angle Measurements

**Figure 4:**
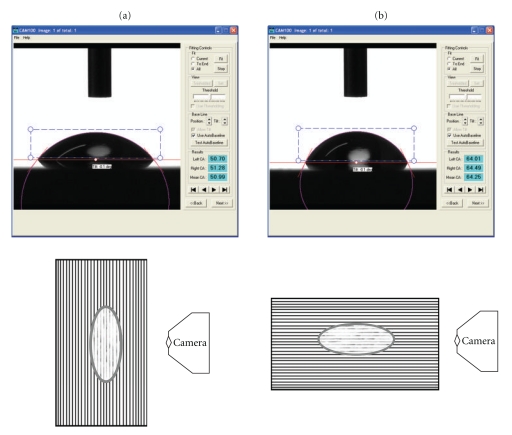
(a) Water contact angle measured by camera with fibers running in-plane (perpendicular) to the camera axis; (b) water contact angle measured by camera with fibers running in-plane (parallel) to the camera axis.

E-glass fiber and BAG-particle composites are hydrophilic materials that show good wettability characteristics.  Table 4 gives the equilibrium contact angles values obtained by the sessile drop method on the different surfaces investigated after mechanical polishing to reach a minimum roughness (i.e., roughness plays no measurable role on contact angle determinations). The contact angle measurements varied on the same FRC surface according to E-glass fiber alignment (Figure 4).

### 3.3. Surface Free Energy Calculations

**Figure 5:**
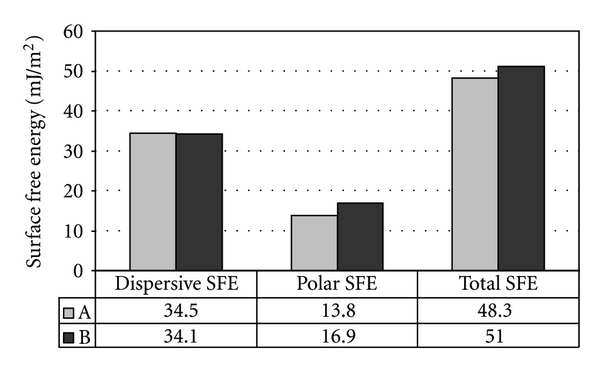
Dispersive (*γ* ^D^), polar (*γ* ^P^), and total (*γ* ^TOT^) components of surface free energy (SFE) calculated using the Owens-Wendt approach: (A) in-plane distribution of fibers; (B) transverse distribution of fibers.

**Figure 6:**
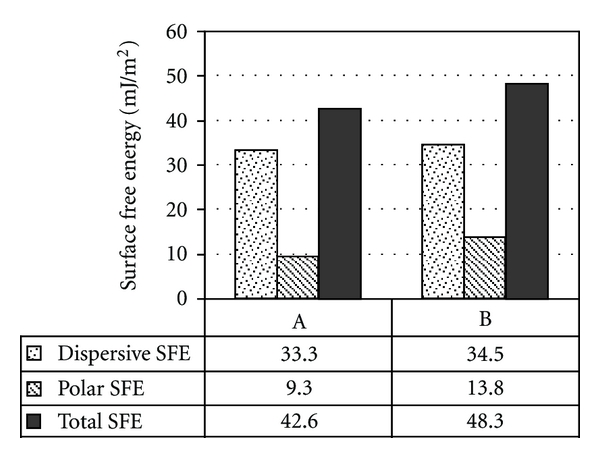
Dispersive (*γ* ^D^), polar (*γ* ^P^), and total (*γ* ^TOT^) components of surface free energy (FRC) calculated using the Owens-Wendt approach: (A) fibers running in-plane (perpendicular) to the camera axis; (B) fibers running in-plane (parallel) to the camera axis.

For the FRC materials (Figure 5), the surface with transverse distribution of fibers (i.e., *θ* = 90°) showed higher (*P* < 0.001) polar (*γ* ^P^) and total SFE (*γ* ^TOT^) components than the surface with in-plane distribution of fibers (i.e., *θ* = 0°). The SFE calculations also varied on the same FRC surface according to E-glass fiber alignment (Figure 6).

**Figure 7:**
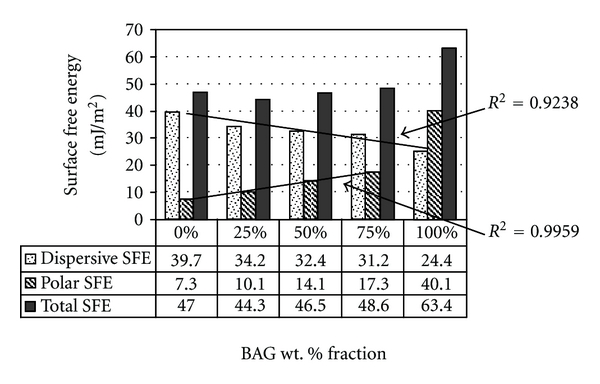
Dispersive (*γ* ^D^), polar (*γ* ^P^), and total (*γ* ^TOT^) components of surface free energy (SFE) for polymer composites of different BAG particles wt. % (0%, 25%, 50%, 75%, and 100%, resp.) calculated using the Owens-Wendt approach.

For the BAG composites (Figure 7), the incremental increase of BAG particle wt. % resulted in an increased (*γ* ^P^) value. In contrast, the dispersive (*γ* ^D^) value decreased, while the total SFE (*γ* ^TOT^) value was of the same order of magnitude for all surfaces.

**Figure 8:**
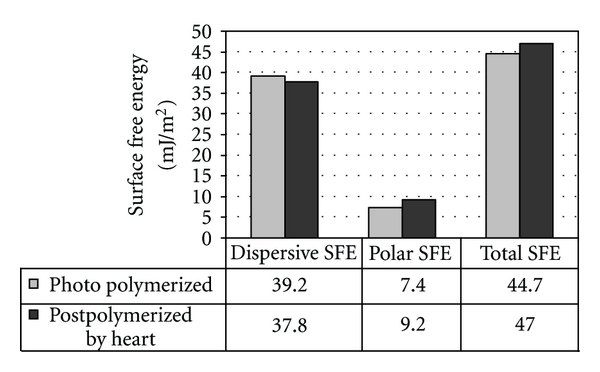
Dispersive (*γ* ^D^), polar (*γ* ^P^), and total (*γ* ^TOT^) components of surface free energy (SFE) for the plain polymer specimens with different polymerization methods calculated using the Owens-Wendt approach.

Postpolymerization by heat improved the SFE components of the specimens (*P* < 0.001). The effect of the curing method on different components of SFE is demonstrated in Figure 8.

The different components of SFE according to the VO approach are not shown. However, the polar component of SFE (*γ* ^P^) determined by the OW approach was always greater than the acidic-basic component (*γ* ^AB^) calculated by the VO approach.

## 4. Discussion

The SFE of a solid surface gives a direct measurement of intermolecular interactions at interfaces and has a strong influence on wetting, adsorption, and adhesion behaviors [36]. SFE and wettability of materials can be determined by measuring the contact angle formed by a range of liquids on a given surface, using several different approaches [18]. The focus of biomaterial development has shifted to the control of wettability of the material surface for improving the attachment of tissues to the implant surface.

There is ongoing research to discover alternative implant materials that could provide better biomechanical matching to the properties of living tissues than titanium affords [23, 37–40]. FRC-BAG composites have shown some interesting results [23]; however, little is known about the effect of exposed E-glass fibers and BAG particles on the surface wettability behavior of polymer. Some animal experiments with implants and their exposed fibers have suggested that glass fibers on the surface of an implant have a positive effect on bone formation [23, 24], and this could be related to the surface wettability. The present study was designed to evaluate whether the glass fiber orientation or the addition of BAG-particles could influence composites surface properties.

### 4.1. Roughness Characterization

The use of a confocal imaging profiler represents an effective methodology for analyzing the surface topography of different biomaterials. The average surface roughness can be qualitatively determined and converted into a numerical reading of the surface topography [41]. The most commonly used dental implant roughness parameter is *R*_*a*_ “the arithmetic medium value of the deviations of the roughness profile in relation to a medium line.” The effect of surface roughening on the resulting contact angles of droplets that reflect the SFE has been studied extensively [9, 11, 12]. Surface roughness will probably account for the contact angle hysteresis on rough surfaces (*R*_*a*_ > 0.1 *μ*m), while the remaining hysteresis on the smooth surfaces (*R*_*a*_ < 0.1 pm) will be caused by surface chemical heterogeneity [12]. In the present study, all the surfaces investigated showed smooth surface characteristics (*R*_*a*_ less than 0.1 *μ*m); therefore, the influence of surface roughness can be considered negligible. Furthermore, it has been clearly demonstrated that surface roughness does not influence the observed contact angles if the equilibrium contact angle on the smooth polymer surface (*R*_*a*_ less than 0.1 *μ*m) is between 60° and 86° [12]. In our study, most of the surfaces investigated showed contact angle values within this given range.

### 4.2. Contact Angle Measurements on Smooth Surfaces

The measuring of contact angles at the solid-air-liquid meeting point is a widely known technique used to investigate the wettability of solid surfaces [42]. The values obtained depend on the surface tension of the liquid, surface topography, cleanliness, and energy of the solid substrate [42, 43]. Thus, the relative wetting characteristics of a liquid-solid interface can be inferred by contact angle measurements.

In this study, SFE calculations were based on contact angle data obtained from three types of liquids: purely nonpolar (Formamide), polar (Diiodomethane), and hydrogen bonded (Water) [44]. The fact that the water contact angles remained within the range of 50° to 65° for all surfaces in general indicates good adhesion and suggests good cell proliferation [45].

Interestingly, as shown in Figure 4, it can be seen that the contact angles are influenced by the orientation of E-glass fibers (i.e., whether the fibers are parallel or perpendicular to camera's axis). We found that when the axis of the camera is perpendicular to the fibers (perpendicular view), the water contact angle values seem to be lower than those obtained when the axis of the camera is parallel to the fibers (parallel view). This could be interpreted by either the function of surface roughness or the function of the SFE of the solid substrate, but as it is mentioned earlier the influence of surface roughness is negligible (*R*_*a*_ less than 0.1 *μ*m); therefore, this phenomena could be more attributed to the influence of the SFE of the exposed E-glass fibers on the substrate surface. Previously, it has been shown that several properties of FRCs, such as mechanical, thermal, and optical properties, are related to the fiber's direction [46–48]. The present study demonstrates that surface wettability is also related to the direction of fibers. The anisotropicity of FRC is typical for many artificial and natural fibrous structures such as wood and bone. Anisotropicity of FRC in terms of wettability is an important observation and should be considered in the fabrication process of FRC devices.

The fact that an increasing wt. % of BAG particles in the polymer matrix improved the wettability may be related to the surface kinetics on the exposed glass particles. From this point of view, the use of BAG particles on the composite implant surfaces seems to be advantageous.

### 4.3. Surface Free Energy Calculations on Smooth Surfaces

Roughness strongly modifies wettability, and if one wants to characterize a substrate surface in terms of surface energy, a smooth surface must be used, because SFE is not a measurement of the topographic morphology of the surface but an indication of the surface tension of the solid surface [11]. Thus, SFE calculations cannot be performed from measurements using a rough surface. Therefore, in the present study, the substrates were polished in order to achieve comparable smooth surface textures. The surface energy is a sum of the polar and dispersive components of surface tension. Many theories and approaches have been proposed for SFE determination. However, formulation of surface and interfacial free energy, as regards its components, is still a debatable issue. In this study, two theoretical models (Owens-Wendt and Van-Oss) were used.

The Owens-Wendt calculations (Figure 6) showed that fiber orientation represents, in fact, a critical issue that must be considered when designing FRC appliances for living tissues.

Interestingly, the BAG composites showed a linear increase in the polar component (*γ* ^P^) but a decrease in the dispersive component (*γ* ^D^) as the wt. % of BAG particles increased. This kept the total component of SFE (*γ* ^TOT^) at the same order of magnitude for all the BAG surfaces. The highest SFE components were found with plain BAG specimens, and this could be interpreted by the absence of polymer within the composite matrix that tends to decrease substrate's surface energy. However, the use of plain BAG material in reconstructive surgeries (i.e., oral implant abutments) is not applicable, whereby the material does not exhibit sufficient mechanical strength under loading conditions.

**Figure 9:**
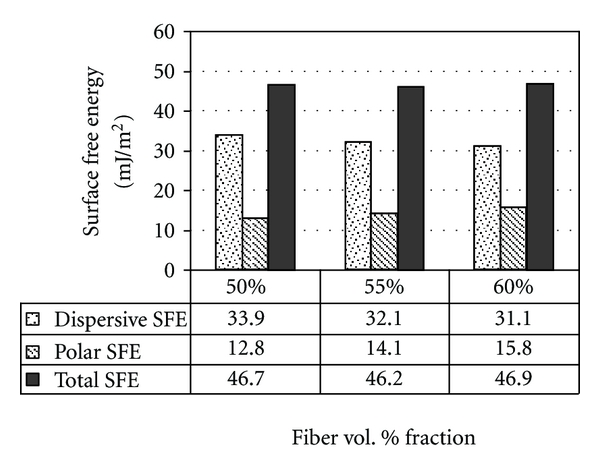
Dispersive (*γ* ^D^), polar (*γ* ^P^), and total (*γ* ^TOT^) components of surface free energy (SFE) for the specimens with different vol. % of E-glass fibers calculated using the Owens-Wendt approach.

Previous work by the authors showed that the mechanical properties of FRC implants are comparable to those of titanium implants and could be tailor-made to fulfill the requirements for several clinical applications [49]. In order to study the effect of fiber density on the surface wettability behavior of FRC implants, the specimens with different fiber vol. % (50%, 55%, and 60%, resp.) were analyzed. Increase in E-glass fiber vol. % had similar effect on surface energy, as did the increasing wt. % of BAG particles (Figure 9).

In both cases, namely, E-glass fibers and BAG, the exposed inorganic glass surface is covered by the hydroxyl group. Hydroxyl-covered substrate or parts of it in the case of composite are the most likely cause of the increased surface wettability of composites. Hydroxylated glass surfaces are also utilized in adhering the glass fibers to the polymer matrix by silane coupling agents [21].

**Figure 10:**
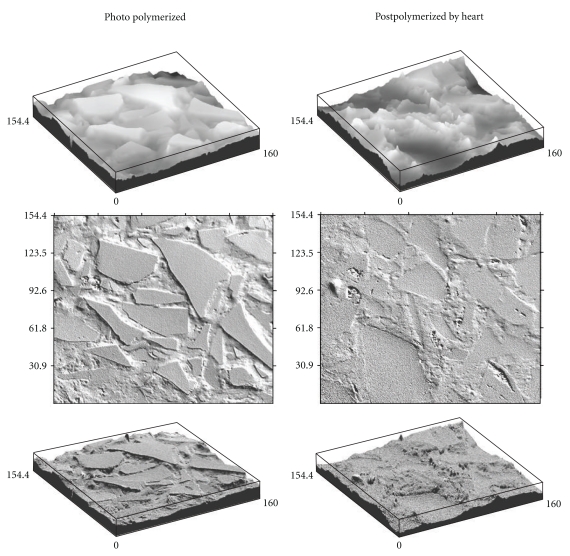
Confocal profiler 3D images of polymer with BAG particles showing the topographical differences between light-cured and oven-cured specimens.

Our finding that postpolymerization heat treatment improved surface hydrophilicity (i.e., smaller contact angle values) is in agreement with earlier reports [50]. MacDougall et al. [30] found that the release of residual monomers from BisGMA-TEGDMA polymer might influence the biocompatibility of polymer implants. As a result of this, composite implants should have an optimum degree of monomer conversion. Ballo et al. [50] have shown that a degree of monomer conversion of approximately 90% of the polymer can be achieved by photopolymerization in a vacuum and postcuring for 24 h at 120°C. This temperature is close to the glass transition temperature (*T*_*g*_) of BisGMA-TEGDMA copolymer. With further storage in water, the residual monomers are leached out from the composite materials, which improves the biocompatibility of the polymer. By postpolymerizing by heat, the polymer matrix seemed to be less ground by the polishing process, which is most likely due to higher cross-linking density, that is, higher DC% than that obtained by photopolymerization only.  Figure 10 demonstrates the topographical changes that could be obtained by optimizing the polymerization process.

Potential biological benefits of the good wettability and high SFE of FRC-BAG materials need to be explored in cell culture conditions and experimental animal models before any definitive conclusions about the importance of fibers orientation or addition of BAG on tissue integration can be drawn.

## 5. Conclusions

Based on the results obtained in this study, the following can be concluded.

1. E-glass fibers and BAG particles composites are hydrophilic materials that show good wettability characteristics towards water.
2. The anisotropic nature of FRC was demonstrated in terms of wettability and SFE.
3. Optimizing the DC % improves the SFE of polymers.

## Figures and Tables

**Table 1:** Materials used in the investigation.

Product	Description	Manufacturer	Lot no.	Composition
E-glass* fiber	Unidirectional fiber	Ahlstrom, Karhula, Finland	11372313	55% SiO_2_, 15% Al_2_O_3_, 22% CaO, 6% B_2_O_3_, 0.5% MgO, and >1.0% Fe + Na + K
Stick resin	Light curing resin	Stick Tech, Turku, Finland	54031672	BisGMA–** TEGDMA*** (50–50%)
BAG**** particles	(S53P4) particles <50 *μ*m	Vivoxid Ltd, Turku, Finland	ABM S53-8-01	SiO_2_ 53 wt. %, Na_2_O 23 wt. %, CaO 20 wt. %, and P_2_O_5_ 4 wt. %

*E-glass: electrical glass, R336 silane sizing.

**BisGMA: bisphenol A-glycidyl dimethacrylate.

***TEGDMA: triethylenglycoldimethacrylate.

****BAG: bioactive glass.

**Table 2:** Test liquids and their surface tension components.

Liquid	Source	Surface tension (mN/m) [16]			
		*γ* ^TOT^	*γ* ^D^	*γ* ^+^	*γ* ^−^
Distilled water, ultrapure water Milli-Q	Produced in-house	72.8	21.8	25.5	25.5
Diiodomethane >99% purity	Sigma-Aldrich, St. Louis, USA Lot # S82251	50.8	50.8	0	0
Formamide, pro analysis	Merck, Darmstadt, Germany Lot #1.09684.2500	58	39	2.28	39.6

**Table 3:** Mean values and standard deviations (SD) of the specimens' surface roughness recorded with a confocal image profiler.

Groups	Mean roughness, *μ*m (SD)	
	Photo polymerized	Postpolymerized by heat
(1) Polymer with in-plane oriented fibers	0.061 (0.013)	0.037 (0.008)
(2) Polymer with transversely oriented fibers	0.024 (0.003)	0.018 (0.004)
(3) 100% polymer (BisGMA-TEGDMA)	0.006 (0.001)	0.008 (0.001)
(4) 75% polymer + 25 wt. % fraction of BAG-particles	0.035 (0.008)	0.051 (0.021)
(5) 50% polymer + 50 wt. % fraction of BAG-particles	0.042 (0.003)	0.096 (0.026)
(6) 25% polymer + 75 wt. % fraction of BAG-particles	0.088 (0.012)	0.045 (0.018)

(7) 100% BAG*	0.012 (0.003)	

*No curing.

**Table 4:** Mean values and standard deviations (SD) of contact angle measurements.

Groups	Contact angle, degrees (SD)					
	Photo polymerized			Postpolymerized by heat		
	Water	Diiodomethane	Formamide	Water	Diiodomethane	Formamide
(1) (A) Polymer with in-plane oriented fibers (perpendicular view)	60.0 (1.4)	45.9 (0.6)	41.0 (1.3)	50.0 (1.0)	44.9 (1.0)	43.4 (0.8)
(B) Polymer with in-plane oriented fibers (parallel view)	69.0 (1.3)	48.0 (0.9)	51.0 (1.2)	65.8 (1.1)	47.1 (0.6)	49.3 (0.9)
(2) Polymer with transversely oriented fibers	54.3 (0.9)	44.6 (0.5)	39.4 (0.3)	52.0 (1.5)	43.2 (0.7)	39.8 (0.8)
(3) 100% polymer (BisGMA-TEGDMA)	70.2 (1.0)	39.8 (0.6)	49.8 (1.4)	65.6 (1.4)	36.7 (0.6)	47.9 (0.5)
(4) 75% polymer + 25 wt. % fraction of BAG-particles	66.4 (1.3)	44.7 (0.9)	50.4 (0.8)	61.7 (1.4)	46.8 (1.0)	48.3 (0.9)
(5) 50% polymer + 50 wt. % fraction of BAG-particles	61.2 (1.5)	50.5 (0.9)	42.5 (0.6)	57.1 (1.2)	49.2 (0.5)	38.2 (0.7)
(6) 25% polymer + 75 wt. % fraction of BAG-particles	55.4 (1.9)	48.8 (1.0)	45.5 (0.6)	51.9 (1.7)	50.7 (1.1)	45.5 (0.6)

(7) 100% BAG*	30.9 (1.9)	67.3 (1.8)	28.4 (3.5)			

*No curing.
